# Identification of hub genes involved in the development of hepatocellular carcinoma by transcriptome sequencing

**DOI:** 10.18632/oncotarget.19483

**Published:** 2017-07-22

**Authors:** Yongchang Zheng, Junyu Long, Liangcai Wu, Haohai Zhang, Lin Li, Ying Zheng, Anqiang Wang, Jianzhen Lin, Xiaobo Yang, Xinting Sang, Ke Hu, Jie Pan, Haitao Zhao

**Affiliations:** ^1^ Department of Liver Surgery, Peking Union Medical College Hospital, Chinese Academy of Medical Sciences & Peking Union Medical College, Beijing, China; ^2^ School of Life Sciences, Center for Synthetic and Systems Biology, Ministry of Education Key Laboratory of Bioinformatics, Collaborative Innovation Center for Diagnosis and Treatment of Infectious Diseases, Tsinghua University, Beijing, China; ^3^ State Key Laboratory of Quality Research in Chinese Medicine, Institute of Chinese Medical Science, University of Macau, Macau, China; ^4^ Department of Radiation Oncology, Peking Union Medical College Hospital, Chinese Academy of Medical Sciences & Peking Union Medical College, Beijing, China; ^5^ Department of Radiology, Peking Union Medical College Hospital, Chinese Academy of Medical Sciences & Peking Union Medical College, Beijing, China

**Keywords:** hub gene, differentially expressed protein-coding genes, development, hepatocellular carcinoma, transcriptome sequencing

## Abstract

Hepatocellular carcinoma (HCC) is a leading cause of cancer-related death. The aim of this study was to identify underlying hub genes and dysregulated pathways associated with the development of HCC using bioinformatics analysis. Differentially expressed protein-coding genes were subjected to transcriptome sequencing in 11 pairs of liver cancer tissue and matched adjacent non-cancerous tissue. Gene ontology (GO) and Kyoto Encyclopedia of Genes and Genomes (KEGG) pathway enrichment analyses were performed, followed by protein-protein interaction (PPI) network construction. Hub genes were identified via centralities analysis and verified using published datasets. In total, 720 significantly differentially expressed protein-coding genes were identified in the samples, including 335 upregulated genes and 385 downregulated genes. The upregulated genes were significantly enriched in cell adhesion, biological adhesion and cell-cell adhesion GO terms under biological process (BP). Conversely, the downregulated genes were significantly enriched in embryonic organ morphogenesis, embryonic organ development and embryonic morphogenesis. The KEGG pathway analysis showed that the upregulated genes were enriched in ECM-receptor interaction and focal adhesion pathways. Furthermore, the downregulated genes were enriched in the ErbB, VEGF and MAPK signaling pathways. The PPI network and centralities analysis suggested that ITGA2 and 12 alternate genes were significant hub genes. These findings improve current understanding of the molecular mechanisms underlying HCC development and may be helpful in identifying candidate molecular biomarkers for use in diagnosing, treating and monitoring the prognosis of HCC.

## INTRODUCTION

Liver cancer is a leading cause of malignancy and mortality worldwide. Hepatocellular carcinoma (HCC), the most common malignancy in the liver, accounts for 85-90% of tumors derived from liver tissue [1]. Despite significant advances in early diagnosis and interventional therapies, there remains a need for novel management methods for advanced HCC [2].

HCC develops through a complicated biological process that involves several genomic changes and various pathogenic molecular mechanisms [3]. The process is slow and involves genomic alterations gradually changing the phenotype of liver cells to produce cellular intermediates that evolve to become cancer cells. Studies of the regulation of gene expression are helpful for understanding the pathogenesis of HCC [4]. RNA sequencing (RNA-Seq) has become useful for examining genetic changes on the whole-genome scale and produces large quantities of data [5]. A comprehensive systematic study of differentially expressed pathways and protein-coding gene interactions can more accurately identify the biological changes that occur during HCC carcinogenesis. In addition, protein-protein interaction (PPI) networks can be used to identify highly connected hub genes that play a key role in maintaining network structure [6]. Consequently, analyzing RNA-Seq data using these bioinformatics methodologies can help predict molecular pathogenesis and identify effective biomarkers of cancer.

HCC is a highly heterogeneous disease, and diverse changes in gene expression contribute to the progression of this cancer [7]. To obtain complete understanding of the alterations in gene expression that occur during HCC, RNA-Seq has been used to identify many key gen es involved in disease progression. However, knowledge of the genetic changes that lead to HCC initiation and progression remains fragmented, and key drivers of carcinogenesis are still unknown, limiting the development of targeted therapy for HCC [8]. Furthermore, the overlap of the most significantly dysregulated genes among multiple studies is very low. Inconsistencies in results are caused by various factors, including measurement errors, small sample sizes and different statistical methods [9]. Therefore, understanding the pathogenesis of this disease remains a major challenge, and many hub genes must still be identified.

In the present study, we identified differentially expressed protein-coding genes from RNA transcriptional profiling performed on 11 paired cancer tissues and adjacent non-cancerous tissues. Then, we conducted GO, KEGG, PPI network and centralities analyses to study and identify changes in pathways and hub genes. The aim of this study was to improve understanding of HCC carcinogenesis by providing information concerning the genetic changes that occur during disease progression and to uncover the expression of biomarkers with potential use for clinical diagnosis, treatment, and monitoring of disease progression.

## RESULTS

### Identification of differentially expressed protein-coding genes in HCC and adjacent non-cancerous tissues

**Figure 1:**
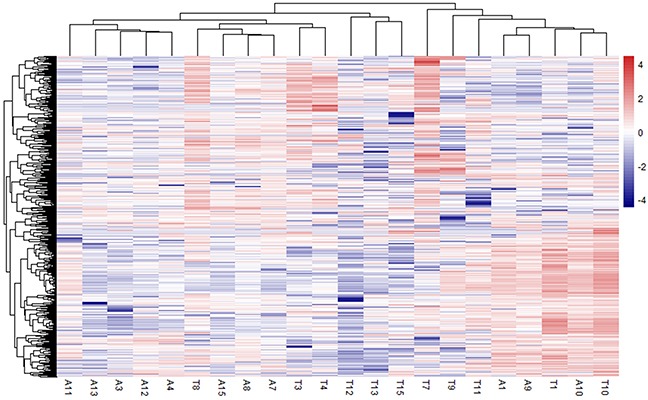
Heatmap showing significantly differentially expressed protein-coding genes among 11 paired HCC and adjacent non-cancerous tissue Rows represent genes, and columns represent samples.

Fifteen pairs of samples from tumor and adjacent tissues were collected from patients enrolled in Peking Union Medical College Hospital from May 2015 to April 2016. All patients provided consent. RNA transcriptional profiling was performed on the tissue samples. Approximately 542.8 million reads were mapped to the human hg38 genome, with a mean of 15.7 M reads per sample (range 4.2-23.5 M) (Supplementary Table 1). Paired samples A2 and T2 were filtered out because of the small amount of data obtained. Multidimensional scaling analysis (MDS) was performed to show that the tumor samples were distinct from the adjacent non-cancerous tissue samples [10]. Four samples (A5, T5, T6, and T14) were found to be potentially misidentified and were excluded from further analysis (Supplementary Figure 1). Hence, transcriptome data from 11 paired samples was used to identify differentially expressed genes. We identified 720 significantly differentially expressed protein-coding genes, including 335 upregulated genes and 385 downregulated genes; a heatmap showing these genes can be found in Figure 1.

### Functional characterization of differentially expressed protein-coding genes

**Figure 2:**
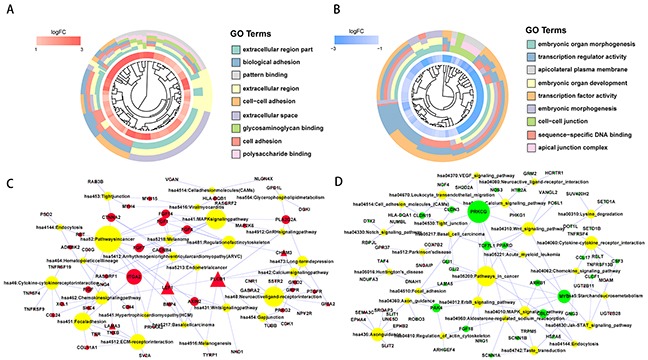
Functional enrichment analysis of significantly upregulated and downregulated protein-coding genes GOcluster plot showing a circular dendrogram of the clustering of the expression spectrum. The inner ring indicates the color-coded logFC. Red represents significantly upregulated **(A)** and blue represents significantly downregulated **(B)** protein-coding genes. The outer ring displays the assigned functional terms. KEGG pathway enrichment of significantly upregulated **(C)** and downregulated **(D)** protein-coding genes. The red node represents significantly upregulated protein-coding genes. The green node represents significantly downregulated protein-coding gene. The yellow node represents enriched pathway symbols. The triangular node represents HCC driver genes from the Driver DB V2 database. The size of the node represents the number of genes.

To gain insight into the functional characteristics of the identified protein-coding genes, we conducted GO and KEGG pathway enrichment analyses [11]. The GO analysis results showed that the upregulated protein-coding genes were significantly enriched in the cell adhesion, biological adhesion and cell-cell adhesion categories under biological process (BP). Under molecular function (MF), the genes were enriched in glycosaminoglycan binding, polysaccharide binding and pattern binding. Additionally, GO cellular component (CC) analysis revealed genes significantly enriched in extracellular region part, extracellular region, and extracellular space. For the downregulated protein-coding genes, for BP, there was significant enrichment in embryonic organ morphogenesis, embryonic organ development and embryonic morphogenesis. For MF, there was enrichment in sequence-specific DNA binding, transcription regulator activity and transcription factor activity. In addition, GO CC analysis revealed genes significantly enriched in cell-cell junction, apical junction complex and apicolateral plasma membrane (Figure 2 and Supplementary Table 2). Moreover, we found upregulated protein-coding genes that were significantly enriched in the ECM-receptor interaction and focal adhesion pathways using KEGG pathway enrichment analysis (Figure 2 and Supplementary Table 3). Meanwhile, downregulated protein-coding genes were enriched in the ErbB, VEGF, and MAPK signaling pathways (Figure 2 and Supplementary Table 3). We then compared these enriched genes with potential HCC driver genes from the Driver DB 2.0 database and identified 15 protein-coding genes that were recorded as HCC driver genes (triangle node) (Figure 2).

### PPI network construction and centralities analysis

**Figure 3:**
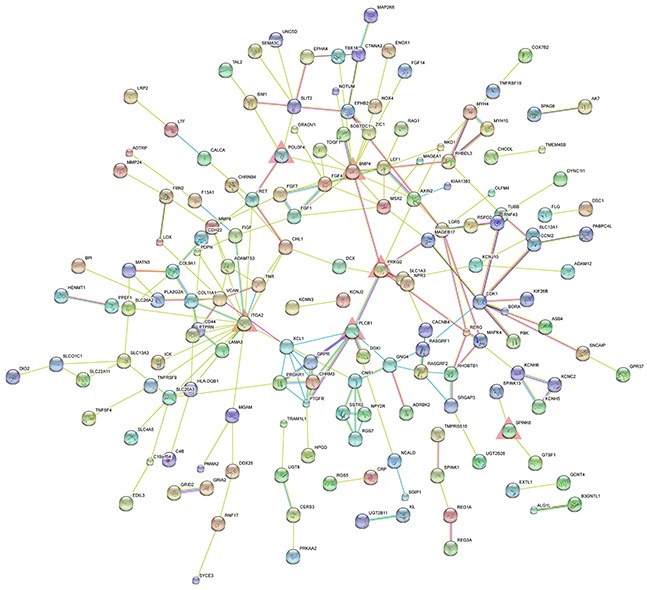
PPI network of significantly upregulated protein-coding genes The nodes represent the significantly upregulated protein-coding genes. The edges represent the interaction of significantly upregulated protein-coding genes. The red triangles represent the significantly upregulated hub genes.

PPI network analysis is a powerful tool for recognizing critical hub members among a cluster of molecules. Thus, we conducted PPI network analysis using the STRING database to identify critical members among our protein-coding genes. The PPI networks for the upregulated and downregulated genes are shown in Figure 3 and Supplementary Figure 2, respectively.

**Figure 4:**
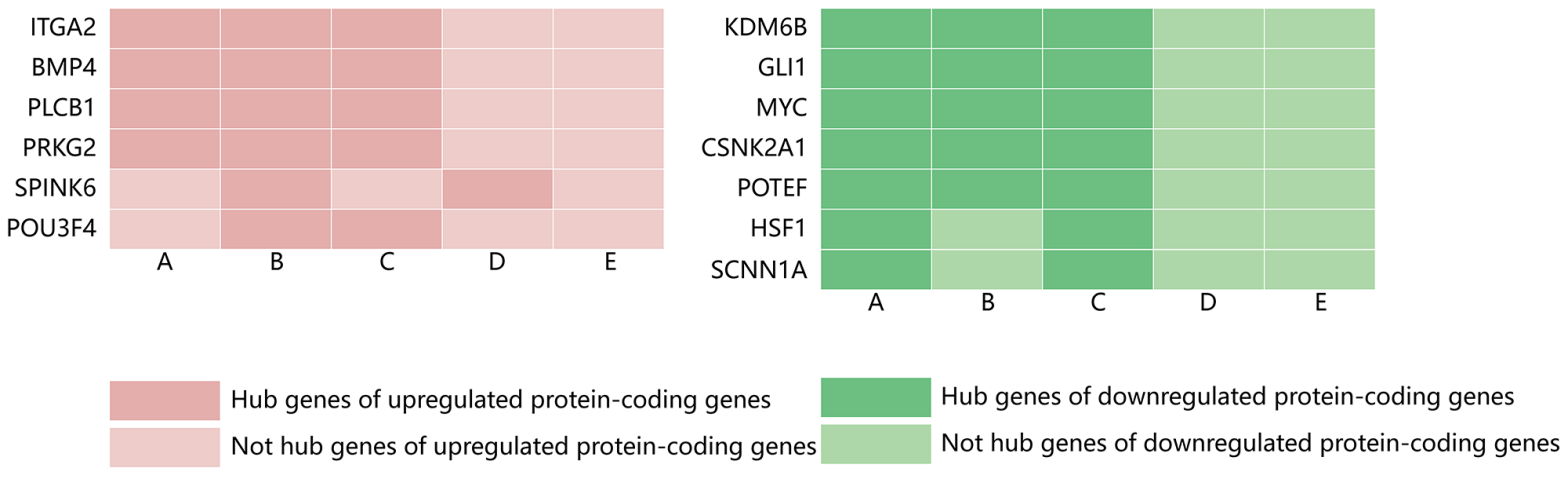
Distribution of hub genes among the significantly upregulated and downregulated protein-coding genes identified by five types of centrality **(A)** Degree centrality; **(B)** betweenness centrality; **(C)** stress centrality; **(D)** closeness centrality; and **(E)** clustering coefficient.

Centralities represent the possibility that a gene is functionally able to keep communicating nodes together in a biological network. There are five types of centralities: degree centrality, betweenness centrality, stress centrality, closeness centrality and clustering coefficient. The five types of centralities were calculated based on the complex network, and we found that the distribution of the top 10 genes was not completely consistent among various centralities analyses. Therefore, the genes that were among the top 10 identified and that were shared more than twice among the five types of centralities were defined as hub genes. Using these criteria, 6 hub genes were obtained for the upregulated protein-coding genes, with the alpha 2 integrin gene (ITGA2), bone morphogenetic protein 4 (BMP4), PLCB1 and PRKG2 identified as the common hub genes across the degree, betweenness and stress centrality analyses. Moreover, SPINK6 and POU3F4 detected in two of the five types of analysis. In addition, 7 hub genes were identified for the downregulated protein-coding genes, with lysine (K)-specific demethylase 6B (KDM6B), GLI1, MYC, CSNK2A1 and POTEF identified as common hub genes across the degree, betweenness and stress centrality analyses, and HSF1 and SCNN1A were commonly obtained in the degree and stress centrality analyses (Figure 4).

### The role of hub genes in the development of HCC

**Figure 5:**
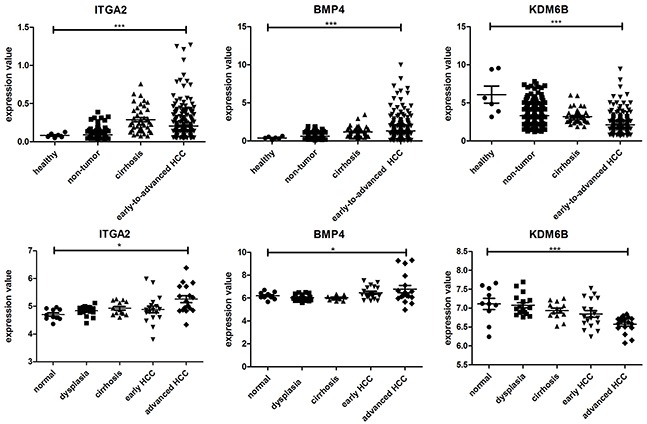
Dynamic expression of ITGA2, BMP4 and KDM6B in HBV-related HCC (up) and HCV-related HCC (down); p < 0. 01 (*), p < 0.001 (**), and p < 0.0001 (***)

To further explore the functional roles of the identified critical hub molecules in the development of HCC, we evaluated changes in the expression spectra of the 13 hub genes using one-way ANOVA (Kruskal-Wallis test) in HBV-related HCC (GSE25097) and HCV-related HCC datasets (GSE6764). We did not identify SCNN1A and PLCB1 in the HBV-related HCC dataset or POTEF and MYC in the HCV-related HCC dataset. The transcriptome profiling data in the GSE25097 dataset contain 557 samples, including 6 healthy livers, 40 cirrhotic tissue samples, 243 adjacent non-tumor samples, and 268 early-to-advanced stage HCC samples. Moreover, the GSE6764 dataset contains a total of 75 tissue samples, including 10 healthy liver samples, 13 cirrhosis samples, 17 dysplastic nodules, 18 early HCC samples and 17 advanced HCC samples. Consistent with our results, ITGA2, BMP4 and PLCB1 were significantly upregulated, and KDM6B and MYC were significantly downregulated during HCC oncogenesis (Figure 5 and Supplementary Figure 4). In contrast to our findings, GLI1, CSNK2A1, POTEF and HSF1 were upregulated and SPINK6 downregulated in the HCC cohort (Supplementary Figures 3 and 4). No significant differences were found in the expression of the other 3 genes (Supplementary Figures 3 and 4). Based on these results, the expression of ITGA2, BMP4 and PLCB1 may contribute to cancer development. ITGA2, BMP4, PLCB1, KDM6B and MYC represent the most likely diagnostic or therapeutic biomarkers associated with HCC.

## DISCUSSION

HCC is a common malignant tumor with a heterogeneous molecular pathogenesis that has not been fully elucidated. Identifying significantly dysregulated genes and pathways associated with HCC carcinogenesis may improve understanding of the molecular pathogenesis underlying HCC development and could identify potential biomarkers for treatment [4]. In this study, GO and KEGG pathway enrichment analysis showed that cell adhesion, biological adhesion and cell-cell adhesion were significant GO terms in upregulated protein-coding genes and that embryonic organ morphogenesis, embryonic organ development and embryonic morphogenesis were significant GO terms in downregulated protein-coding genes. Moreover, ECM-receptor interactions and focal adhesion pathways were the relevant pathways associated with the upregulated protein-coding genes, and the ErbB, VEGF, and MAPK signaling pathways were the relevant pathways associated with the downregulated protein-coding genes.

Hub genes were identified based on degree centrality, betweenness centrality, stress centrality, closeness centrality and clustering coefficients in the PPI network. The outcomes across these five centralities were integrated to address inconsistent results provided by the different methods of analysis. A total of 6 hub genes were identified among the upregulated protein-coding genes, and 7 hub genes were obtained among the downregulated protein-coding genes. ITGA2, BMP4, PLCB1, PRKG2, KDM6B, GLI1, MYC, CSNK2A1 and POTEF were common hub genes across three centrality methods. In addition, the expression spectra of the 13 hub genes were verified in HCC tumorigenesis using GEO datasets GSE25097 and GSE6764. ITGA2, BMP4, and PLCB1 were significantly upregulated, and KDM6B and MYC were significantly downregulated during HCC oncogenesis, consistent with our results. In contrast, GLI1, CSNK2A1, POTEF and HSF1 were upregulated and SPINK6 downregulated in the HCC cohort. No significant differences were found in the expression of the other 3 genes. BMP4 and ITGA2 were identified as critical hub molecules among the upregulated protein-coding genes and had many interactions with their neighbors. KMD6B was considered to be a hub molecule due to its high degree of downregulation when compared to all protein coding genes. Therefore, we mainly discuss the function of these top degree centrality genes.

KDM6B is a demethylase that acts on histone H3 at lysine 27 (H3K27), which specifically catalyzes the demethylation of H3 lysine-27 tri/di methylation (H3K27me3/2) [12]. KDM6B has been shown to play a key role in promoting transcription elongation associated with related elongation factors and RNA polymerase II to activate gene transcription [13, 14]. Growing evidence suggests that KDM6B regulates cancer development. However, the effect of KDM6B on tumorigenesis is not consistent among different human cancers. Compared with adjacent normal tissues, KDM6B expression was significantly increased in renal cell carcinoma [15]. Overexpression of KDM6B has also been shown to promote invasion-metastasis cascades and induce the expression of mesenchymal genes in breast cancer [16]. In contrast, KDM6B knockdown promotes tumor sphere formation, increases hepatic metastasis and peritoneal dissemination in pancreatic adenocarcinoma, and enhances epithelial-mesenchymal transition and invasiveness in colon cancer cells [17, 18]. Similarly, the knockdown of KDM6B inhibits cell apoptosis and promotes cell growth by reducing the nuclear translocation of FOXO1 in non-small cell lung cancer cells [19]. However, literature focused on KDM6B expression in HCC is lacking. In this study, KDM6B was identified as a hub gene among the downregulated protein-coding genes differentially expressed throughout HCC development.

The integrin family of α/β heterodimeric transme-mbrane receptors mediates the binding of cells to the extracellular matrix (ECM) and the intracellular signaling events that occur within the ECM [20]. ITGA2 encodes the integrin α2 subunit of the α2β1 integrin receptor, which is a cell adhesion molecule [20]. Previous studies have shown that the ITGA2 gene is associated with various types of cancer, including colorectal cancer, prostate cancer, hepatocellular carcinoma, pancreatic cancer, breast cancer, melanoma and ovarian carcinoma. ITGA2 is expressed in colon cancer cell lines and is involved in the proliferation and migration of these cells [21]. Slambrouk *et al*. reported that integrin α2 subunits interact with the ECM protein collagen I, increasing prostate cancer cell adhesion and cellular invasion [22]. Integrin α2 subunits also downregulate E-cadherin-mediated cell-cell adhesion architecture, enhancing pancreatic cancer cell invasiveness, migration and proliferation [23]. In addition, integrin α2β1 inhibits mammalian sterile 20-like kinase 1 kinase (MST1) phosphorylation and activates Yes-associated protein (YAP) oncogenic signaling in HCC [24]. It has been previously shown that ITGA2 is enriched in ECM-receptor interactions and pathways in cancer. However, the impact of ITGA2 on the progression of HCC is still unknown. Therefore, it seems necessary to investigate and clarify the basic biological links between ITGA2 and HCC.

BMP4 belongs to the transforming growth factor (TGF-β) superfamily of extracellular signaling molecules, which is of great relevance both during development and in adult tissues [25, 26]. BMP signaling regulates early liver development and promotes liver bud morphogenesis as well as the migration, proliferation and survival of hepatoblasts [27, 28]. Interestingly, BMP4 has been shown to participate in human carcinogenesis. Recent findings have revealed that BMP4 is overexpressed in breast cancer and may promote cell invasion and migration by modulating TGF-β factor signaling [29]. In addition, BMP4 signaling causes direct overexpression of ID3, a proto-oncogene that contributes to the pathogenesis of human ovarian cancer [30]. BMP4 is upregulated in HCC, and the overexpression of BMP4 promotes the metastasis and proliferation of HCC cells by activating the mitogen-activated protein/extracellular signal-regulated kinase (MEK)/extracellular signal-regulated kinase (ERK) signaling pathway [31]. BMP4 was enriched in KEGG pathways associated with pathways in cancer and basal cell carcinoma in this study. Therefore, BMP4 and its associated pathways may be used as a biological indicator for the development of various types of cancers, including HCC.

Although we identified and verified hub genes important for the development of HCC using comprehensive bioinformatics technology, there are some limitations to the present study. First, this study lacked experimental validation of genes and their functions in HCC carcinogenesis. Second, we cannot rule out the possibility that these key genes may be involved in non-developmental aspects of HCC. Finally, the sample size for the RNA-Seq was small, which may have led to a high rate of false-positive outcomes. Hence, a larger sample size is needed for further bioinformatics analysis, and experimental studies are necessary to validate our results.

In conclusion, we identified several hub genes and systematically presented the biological processes and signaling pathways associated with the development of HCC. Many of these genes were not previously reported but could play important roles in HCC. Further research is required to focus on the clinical application of these genes and pathways for diagnosing, treating and monitoring the prognosis of HCC.

## MATERIALS AND METHODS

### Ethics

This study was approved by the Clinical Research Ethics Committee of Peking Union Medical College Hospital. Written informed consent with a signature was obtained from each patient.

### Clinical samples

We collected tissue samples from patients with liver cancer undergoing surgery at Peking Union Medical College Hospital, Beijing. The samples were collected in pairs, i.e., cancer tissue and adjacent non-cancerous tissue. The collected tissue samples were stored in liquid nitrogen.

### RNA preparation and sequencing

First, 50 mg of tissue was lysed in TRIzol (Invitrogen) to extract RNA following the manufacturer's instructions. Next, ribosomal RNA was depleted using a RiboZero Gold kit (Epicentre Bio-technologies). RNA integrity was assessed with an Agilent Bioanalyzer 2100. An RNA-Seq library was generated with the rRNA-depleted samples using an Illumina standard RNA Sample Prep kit according to the manufacturer's instructions. The library was subsequently sequenced on an Illumina HiSeq2500 as 125-bp paired-ends with approximately 300-bp size selection.

### Published dataset and database

Liver cancer-related RNA-Seq data are available from the NCBI GEO database, including two independent microarray datasets: HBV-related HCC (GSE25097) and HCV-related HCC (GSE6764). HCC-related driver genes were obtained from the Driver DB 2.0 database [32].

### Transcriptome sequencing analysis

Sequencing quality was assessed with FASTQC [33]. After removing adaptor and low-quality reads using cutadapt [34] (-q 10--quality-base=32 -e 0.1 -O 10 -m 50), the clean reads were aligned to human (hg38) GRCh38.p5 (http://www.ncbi.nlm.nih.gov/assembly/GCF_000001405.31) genome reference sequences using Tophat2 [35] (-a 6 --microexon-search -m 2); bam files were generated and sorted, and duplicate reads were then removed using Samtools [36]. Read counts were tabulated with HT-Seq [37] using “union” mode and the Gencode human v24 GTF file as a reference. edgeR was used to identify the small library size, make MDS plots of the samples, and check reproducibility from replicates to remove small sample sizes and outliers and check for batch effects [38]. Significantly differential gene expression between tumor and adjacent non-cancerous tissue was estimated, with a minimum twofold change and FDR less than 0.01, using the edgeR package from Bioconductor. A heatmap was plotted using the pheatmap package. Cufflinks [39] (v2.21) was also used to estimate the total transcriptional output based on the Gencode gene annotation for human HG38 (version 24) [40].

### Gene ontology and KEGG pathway enrichment analysis

We performed Gene Ontology and KEGG pathway enrichment analysis using The Database for Annotation, Visualization and Integrated Discovery (DAVID) version 6.7 (https://david-d.ncifcrf.gov/home.jsp) [11]. Unique lists of significantly differentially expressed protein-coding genes and all the expressed genes (FPKM >0 in any sample) were submitted to the web interface as the gene list and background, respectively. Enrichment results were visualized using R and Cytoscape 3.5.0 software [41]. In addition, we compared these enriched genes with potential HCC driver genes from the Driver DB 2.0 database [32].

### PPI network analysis

Proteins rarely perform their functions independently, and it is therefore important to investigate protein interactions by studying larger functional groups [42]. Genes that were identified as significantly upregulated or downregulated were mapped to the STRING (Search Tool for the Retrieval of Interacting Genes) version 10.0 database (http://www.string-db.org/) and used to evaluate PPI information and construct a PPI network [6]. The STRING database covers 9.6 million proteins from 2031 organisms. In the PPI network, each node represents a gene, and the edges stand for interactions between nodes.

### Centralities analysis of the PPI network

Researchers have revealed strong correlations between PPI networks and the functions of protein/gene components [43]. Topological centrality is effective for identifying molecules that may play important roles in significantly perturbed networks [44]. The PPI data were downloaded from the STRING database. Centralities analysis was performed using Cytoscape 3.5.0 software. We presented the centralities of the PPI network on a local (degree and clustering coefficient) and global scale (betweenness, closeness and stress). Genes identified in the top 10 genes and that were shared more than twice among the five types of centralities were defined as hub genes.

### Data submission

Sequence data has been deposited at the European Genome-phenome Archive (EGA) (https://ega-archive.org), which is hosted by European Bioinformatics Institute (EBI) and the Centre for Genomic Regulation (CRG), under accession number EGAS00001002526.

## SUPPLEMENTARY MATERIALS FIGURES AND TABLES




