# Supplementary material for: Identification of hub genes involved in the development of hepatocellular carcinoma by transcriptome sequencing
Source: Oncotarget. 2017 Jul 22;8(36):60358–67. doi: 10.18632/oncotarget.19483 (PMC5601144; doi:10.18632/oncotarget.19483)
Supplement: Supplementary file 1 [file oncotarget-08-60358-s001.pdf]

Identification of hub genes involved in the development of hepatocellular carcinoma by transcriptome sequencing

SUPPLEMENTARY MATERIALS

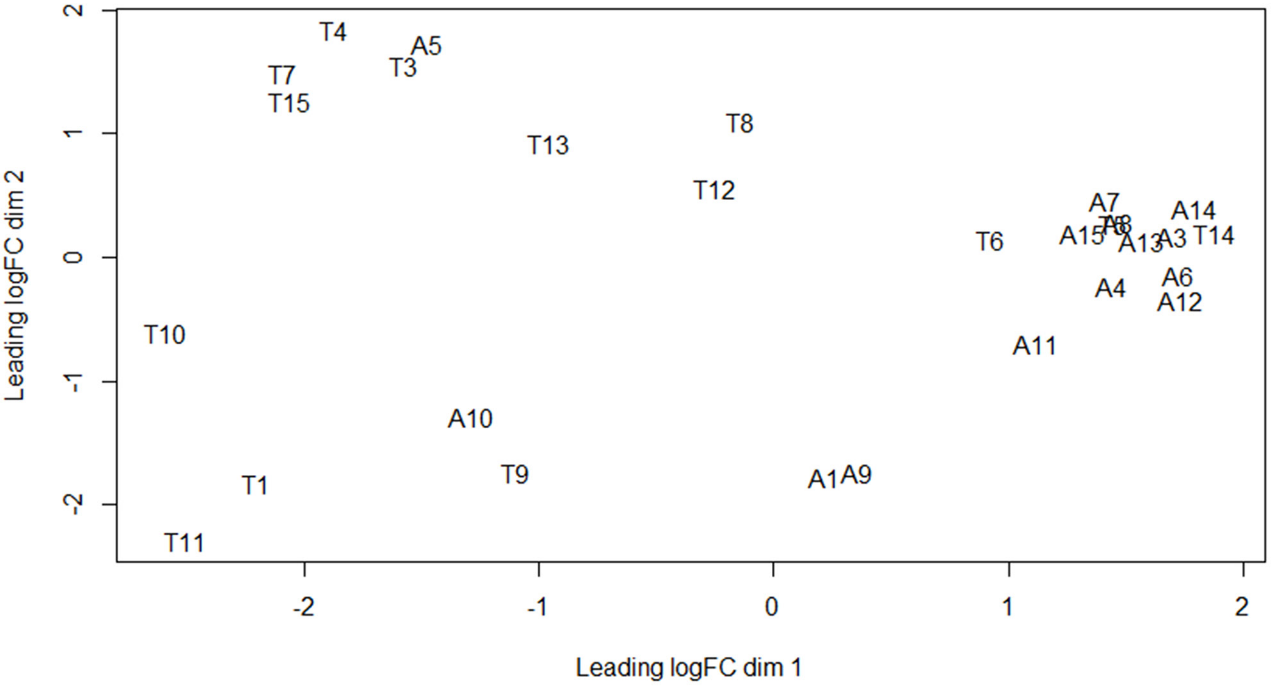

Supplementary Figure 1: Multidimensional scaling (MDS) of HCC-related genes in different samples. MDS displays some mislabeled samples, which includes 4 samples (A5, T5, T6, and T14).

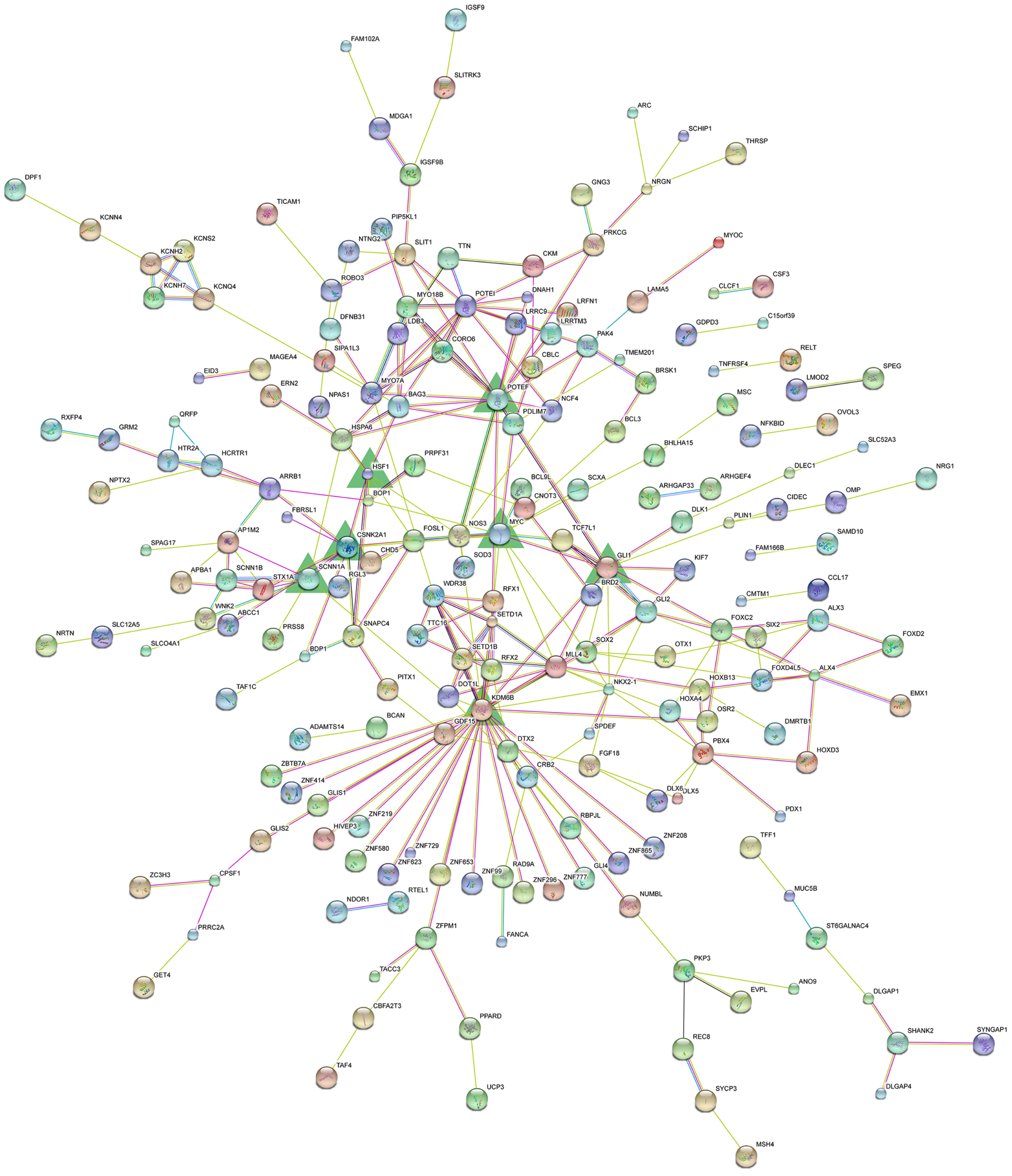

Supplementary Figure 2: Protein-protein interaction network of significantly downregulated protein-coding genes. The nodes represent the significantly downregulated protein-coding genes. The edges represent the interactions of significantly downregulated protein-coding genes. The green triangles represent the significantly downregulated hub genes.

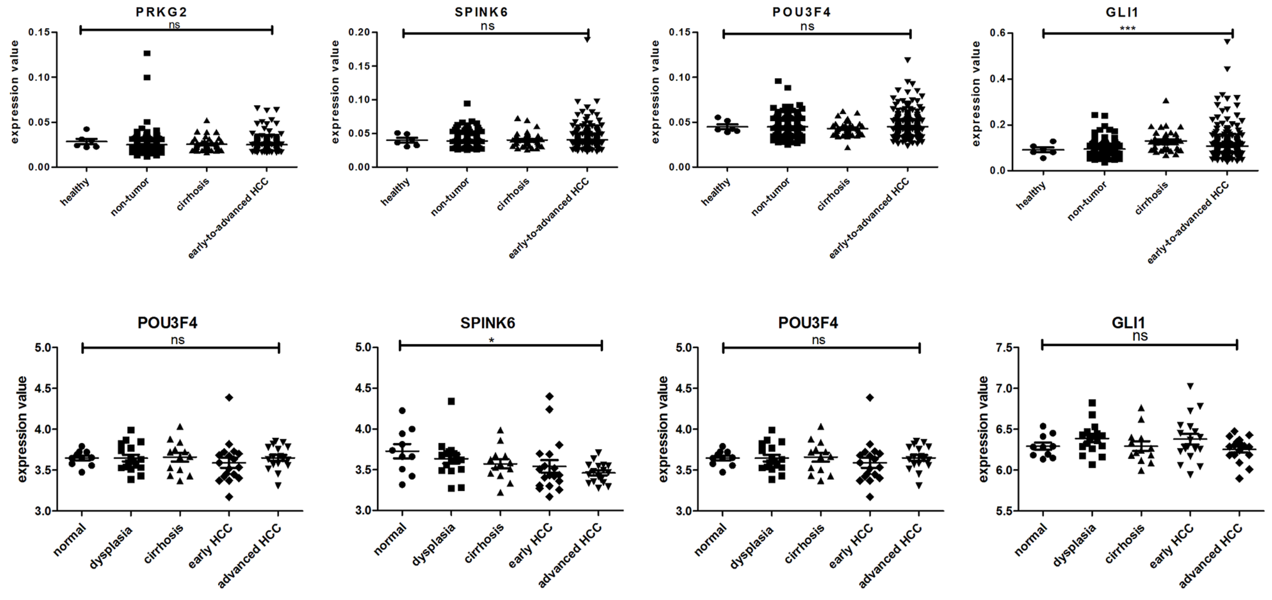

Supplementary Figure 3: Dynamic expression of PRKG2, SPINK6, POU3F4 and GLI1 in HBV-related HCC (up) and HCV-related HCC (down); $p < 0.01$ (*), $p < 0.001$ (**), and $p < 0.0001$ (***).

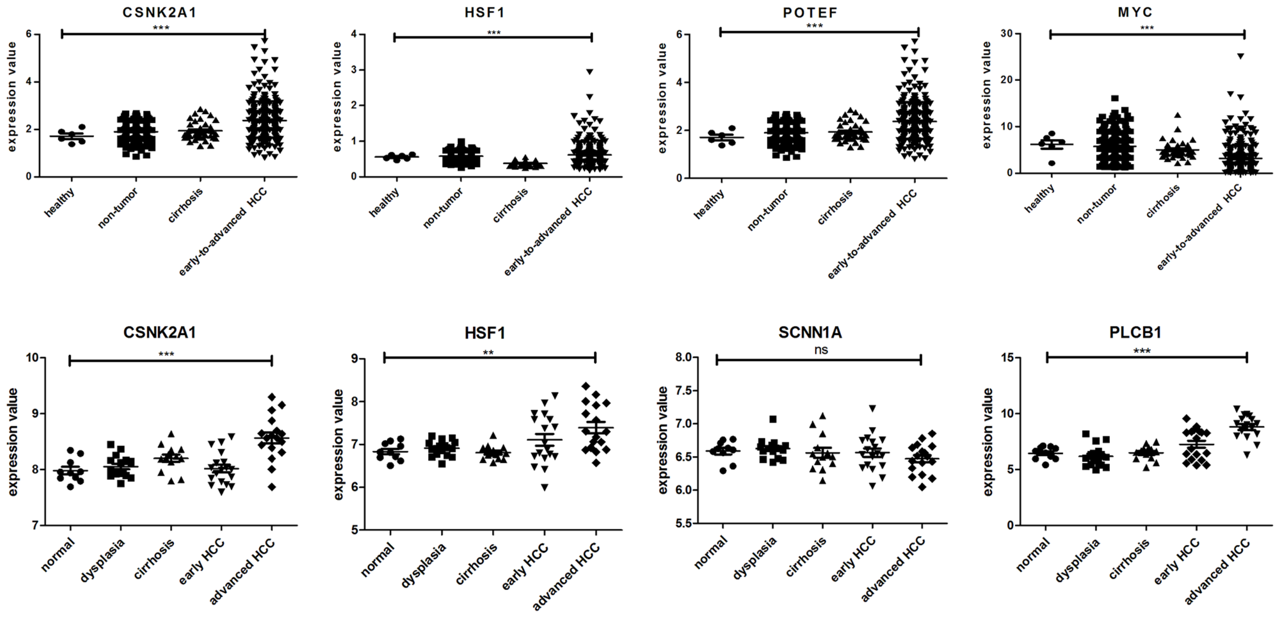

Supplementary Figure 4: Dynamic expression of CSNK2A1, HSF1, POTEF, MYC, SCNN1A and PLCB1 in HBV-related HCC (up) and HCV-related HCC (down); $p < 0.01$ (*), $p < 0.001$ (**), and $p < 0.0001$ (***).

Supplementary Table 1: Overview of the transcriptome in the liver samples

Samples	Raw reads	Mapped (%)	Duplicates removed	Protein coding RNAs (%)	LncRNAs (%)
A1	17948509	84.2	5714372	94.72	3.65
T1	15883949	82.0	5820812	95.22	3.32
A2	4840183	87.6	1839059	95.69	3.22
T2	5549694	89.7	2546454	95.80	3.22
A3	21187487	94.9	6138480	95.65	3.21
T3	24177621	93.5	9361910	96.91	2.31
A4	24882280	90.8	6572384	96.37	2.66
T4	25801068	91.6	8274817	96.56	2.56
A5	21538596	92.0	7232772	95.75	3.13
T5	24324041	93.0	7403792	96.08	2.92
A6	24521590	92.3	7216892	95.25	3.53
T6	24339558	92.9	7266647	95.65	3.22
A7	24117654	93.3	7461649	96.16	2.81
T7	21893007	94.1	8950417	95.60	3.33
A8	24845131	94.2	8451369	96.25	2.71
T8	24047323	93.5	9457445	96.72	2.42
A9	13128558	83.3	4313118	94.59	3.78
T9	16912464	84.3	5951490	95.19	3.44
A10	11862367	81.0	3609672	95.43	3.12
T10	24265065	78.2	5808213	94.56	3.65
A11	19111827	86.6	6315606	97.16	2.04
T11	16071621	86.7	4523902	93.42	4.76
A12	24775524	94.5	5451222	95.75	3.06
T12	24956999	95.0	4688961	96.01	2.94
A13	23171316	94.7	5159147	96.27	2.68
T13	25735081	94.9	5410995	96.67	2.43
A14	25073305	95.0	5152678	96.23	2.71
T14	25214197	94.8	4074400	96.19	2.75
A15	25553790	94.5	4833710	96.02	2.88
T15	25712581	94.6	5554565	96.06	2.93

T: cancer tissue, A: adjacent non-cancerous tissue.

Supplementary Table 2: Gene ontology functional enrichment analysis for significantly upregulated and downregulated protein-coding genes

Category	Term	Gene function	Gene count	P value
Upregulated protein-coding genes				
GOTERM_BP_FAT	GO:0007155	cell adhesion	29	2.21E-05
GOTERM_BP_FAT	GO:0022610	biological adhesion	29	2.27E-05
GOTERM_BP_FAT	GO:0016337	cell-cell adhesion	15	2.71E-04
GOTERM_MF_FAT	GO:0005539	glycosaminoglycan binding	11	0.000124334
GOTERM_MF_FAT	GO:0030247	polysaccharide binding	11	0.000271287
GOTERM_MF_FAT	GO:0001871	pattern binding	11	0.000271287
GOTERM_CC_FAT	GO:0044421	extracellular region part	45	3.61E-09
GOTERM_CC_FAT	GO:0005576	extracellular region	67	1.83E-07
GOTERM_CC_FAT	GO:0005615	extracellular space	30	1.10E-05
Downregulated protein-coding genes				
GOTERM_BP_FAT	GO:0048562	embryonic organ morphogenesis	20	2.44E-12
GOTERM_BP_FAT	GO:0048568	embryonic organ development	21	3.08E-11
GOTERM_BP_FAT	GO:0048598	embryonic morphogenesis	24	6.77E-09
GOTERM_MF_FAT	GO:0043565	sequence-specific DNA binding	31	1.44E-06
GOTERM_MF_FAT	GO:0030528	transcription regulator activity	54	5.78E-06
GOTERM_MF_FAT	GO:0003700	transcription factor activity	40	6.52E-06
GOTERM_CC_FAT	GO:0005911	cell-cell junction	9	0.011585712
GOTERM_CC_FAT	GO:0043296	apical junction complex	5	0.074824629
GOTERM_CC_FAT	GO:0016327	apicolateral plasma membrane	5	0.081483335

Supplementary Table 3: Pathway functional enrichment analysis for significantly upregulated and downregulated protein-coding genes

See Supplemantary File 1
