# Supplementary material for: Identification of hub genes involved in the development of hepatocellular carcinoma by transcriptome sequencing
Source: Oncotarget. 2017 Jul 22;8(36):60358–67. doi: 10.18632/oncotarget.19483 (PMC5601144; doi:10.18632/oncotarget.19483)
Supplement: Supplementary file 2 [file oncotarget-08-60358-s002.docx]

**Supplementary Table 3: Pathway functional enrichment analysis for significantly upregulated and downregulated protein-coding genes.**

| **Term** | **Name** | **Gene count** | **P value** |
| --- | --- | --- | --- |
| Upregulated protein-coding genes |  |  |  |
| hsa04512 | ECM-receptor interaction | 7 | 0.002780712 |
| hsa05200 | Pathways in cancer | 12 | 0.021379683 |
| hsa04730 | Long-term depression | 5 | 0.028678583 |
| hsa04510 | Focal adhesion | 8 | 0.052520553 |
| hsa04360 | Axon guidance | 6 | 0.066390752 |
| hsa04080 | Neuroactive ligand-receptor interaction | 9 | 0.066413753 |
| hsa05412 | Arrhythmogenic right ventricular cardiomyopathy (ARVC) | 4 | 0.136852559 |
| hsa00500 | Starch and sucrose metabolism | 3 | 0.158074253 |
| hsa04540 | Gap junction | 4 | 0.190235057 |
| hsa04062 | Chemokine signaling pathway | 6 | 0.208611874 |
| hsa05213 | Endometrial cancer | 3 | 0.219356242 |
| hsa05217 | Basal cell carcinoma | 3 | 0.238172237 |
| hsa00564 | Glycerophospholipid metabolism | 3 | 0.320034827 |
| hsa05416 | Viral myocarditis | 3 | 0.338739366 |
| hsa04530 | Tight junction | 4 | 0.396486648 |
| hsa05410 | Hypertrophic cardiomyopathy (HCM) | 3 | 0.423557668 |
| hsa04640 | Hematopoietic cell lineage | 3 | 0.429417339 |
| hsa04060 | Cytokine-cytokine receptor interaction | 6 | 0.457566632 |
| hsa04310 | Wnt signaling pathway | 4 | 0.473174915 |
| hsa04912 | GnRH signaling pathway | 3 | 0.497176559 |
| hsa04916 | Melanogenesis | 3 | 0.502595188 |
| hsa04020 | Calcium signaling pathway | 4 | 0.577281182 |
| hsa05012 | Parkinson's disease | 3 | 0.642896152 |
| hsa04514 | Cell adhesion molecules (CAMs) | 3 | 0.659639503 |
| hsa04144 | Endocytosis | 3 | 0.82476127 |
| hsa04010 | MAPK signaling pathway | 9 | 0.080548873 |
| hsa05218 | Melanoma | 4 | 0.117940325 |
| hsa04810 | Regulation of actin cytoskeleton | 6 | 0.29825178 |
| Downregulated protein-coding genes |  |  |  |
| hsa00310 | Lysine degradation | 4 | 0.024689866 |
| hsa04012 | ErbB signaling pathway | 5 | 0.035606629 |
| hsa04742 | Taste transduction | 4 | 0.03799776 |
| hsa04310 | Wnt signaling pathway | 6 | 0.064750632 |
| hsa05200 | Pathways in cancer | 9 | 0.096174644 |
| hsa04960 | Aldosterone-regulated sodium reabsorption | 3 | 0.116608055 |
| hsa04330 | Notch signaling pathway | 3 | 0.14570209 |
| hsa05217 | Basal cell carcinoma | 3 | 0.186464995 |
| hsa05221 | Acute myeloid leukemia | 3 | 0.202154724 |
| hsa04670 | Leukocyte transendothelial migration | 4 | 0.239586388 |
| hsa04370 | VEGF signaling pathway | 3 | 0.292793341 |
| hsa04060 | Cytokine-cytokine receptor interaction | 6 | 0.322923989 |
| hsa04630 | Jak-STAT signaling pathway | 4 | 0.385328083 |
| hsa04020 | Calcium signaling pathway | 4 | 0.46629129 |
| hsa04010 | MAPK signaling pathway | 5 | 0.539001791 |
| hsa04360 | Axon guidance | 3 | 0.557374404 |
| hsa04514 | Cell adhesion molecules (CAMs) | 3 | 0.569992341 |
| hsa04530 | Tight junction | 3 | 0.578262156 |
| hsa05016 | Huntington's disease | 3 | 0.737279193 |
| hsa04144 | Endocytosis | 3 | 0.748405197 |
| hsa04062 | Chemokine signaling pathway | 3 | 0.756487021 |
| hsa04510 | Focal adhesion | 3 | 0.791344433 |
| hsa04810 | Regulation of actin cytoskeleton | 3 | 0.821797705 |
| hsa04080 | Neuroactive ligand-receptor interaction | 3 | 0.889582716 |
